# Olink proteomics analysis uncovers the landscape of inflammation-related proteins in patients with acute compartment syndrome

**DOI:** 10.3389/fimmu.2023.1293826

**Published:** 2023-11-17

**Authors:** Tao Wang, Shuo Yang, Yubin Long, Yiran Li, Ting Wang, Zhiyong Hou

**Affiliations:** ^1^ Department of Orthopaedic Surgery, Third Hospital of Hebei Medical University, Shijiazhuang, Hebei, China; ^2^ Orthopaedic Research Institute of Hebei Province, Shijiazhuang, Hebei, China; ^3^ Department of Orthopedics, The First Central Hospital of Baoding, Baoding, China; ^4^ Department of Nursing, Third Hospital of Hebei Medical University, Shijiazhuang, Hebei, China

**Keywords:** tibial fractures, proteomics, cytokines, inflammation, CCL23, CSF-1, IL6 and HGF

## Abstract

**Purpose:**

Our primary purpose was to explore the landscape of inflammation-related proteins, and our second goal was to investigate these proteins as potential biomarkers of acute compartment syndrome (ACS), which is a serious complication of tibial fractures.

**Methods:**

We collected sera from 15 healthy subjects (control group, CG) and 30 patients with tibial fractures on admission day, comprising 15 patients with ACS (ACS group, AG) and 15 patients without ACS (fracture group, FG). Ten samples in each group were analyzed by the inflammation panel of Olink Proteomics Analysis, and all samples were verified by an ELISA. Receiver-operating characteristic (ROC) curve analysis was performed to identify the diagnostic ability and cutoff values of potential biomarkers.

**Results:**

Our findings showed that the levels of IL6, CSF-1, and HGF in the FG were significantly higher than those in the CG. Similar results were found between the AG and CG, and their cutoff values for predicting ACS compared with the CG were 9.225 pg/ml, 81.04 pg/ml, and 0.3301 ng/ml, respectively. Furthermore, their combination had the highest diagnostic accuracy. Notably, compared with FG, we only found a higher expression of CCL23 in the AG. Additionally, we identified 35.75 pg/ml as the cutoff value of CCL23 for predicting ACS in patients with tibial fractures.

**Conclusion:**

We identified CCL23 as a potential biomarker of ACS in comparison with tibial fracture patients and the significance of the combined diagnosis of IL6, CSF-1, and HGF for predicting ACS compared with healthy individuals. Furthermore, we also found their cutoff values, providing clinicians with a new method for rapidly diagnosing ACS. However, we need larger samples to verify our results.

## Introduction

Acute compartment syndrome (ACS) is a severe orthopedic emergency that affects 1–30.4% of patients with lower extremity fractures, based on previous reports (1–3). It is characterized by rapidly increased pressure within a closed compartment after trauma, causing bleeding, tissue edema, and reduced limb perfusion. Its diagnosis is commonly based on clinical symptoms, such as pain, pain with passive stretching, and swelling, and the experience of clinicians rather than gold standard tests or biomarkers. A delay in diagnosis or treatment can result in unimaginable outcomes, such as sensory deficits, paralysis, infection, or muscle necrosis (4–6). Our latest studies have reported that neutrophils (NEU) and creatine kinase myocardial bands (CKMB) may be biomarkers of ACS (2, 7). However, there was a limited number of complete blood counts and derived inflammatory indicators. Thus, biomarkers for predicting ACS are urgently needed.

Proteomics techniques have greatly advanced over the last decade, and there has been an increase in the number of protein assays utilizing a variety of detection techniques, such as fluorescence and polymerase chain reaction (8). Recently, Olink technology has been widely used due to its excellent reproducibility and stability (8, 9). Additionally, it not only offers various assay panels targeted toward different diseases but also requires small sample volumes, which is crucial for the limited number of clinical samples (9). The improvement of proteomics technologies will expand our understanding of diseases, identify biomarkers of clinical value, and ultimately contribute to human health. Previous studies have reported that inflammation is a major driving factor in the development of ACS (10). To our knowledge, no previous study has reported Olink Proteomics Analysis in ACS patients. Therefore, our primary purpose is to explore the landscape of inflammation-related proteins, and our second goal is to investigate these proteins as potential biomarkers.

## Materials and methods

### Ethics approval and consent to participate

This study was approved by the Institutional Review Board of our hospital before data collection. Informed consent forms from patients and healthy individuals were obtained.

### Subjects

This study was conducted in our hospital from July 2022 to July 2023, and was approved by the hospital’s ethics committee (No. K2020-024-1). We collected serum samples from 15 healthy subjects and 30 tibial fracture patients, comprising 15 patients without ACS and 15 patients with ACS. They were divided into a control group (CG), fracture group (FG), and ACS group (AG), respectively. Finally, we randomly selected ten samples in each group to perform the inflammation panel of Olink Proteomics Analysis, and then all samples were verified by an ELISA.

The inclusion criteria for tibial fracture patients were as follows: 1) 18–65 years old; 2) closed tibial fracture; and 3) diagnosed with ACS based on the ΔP<30mmHg (ΔP=diastolic arterial pressure-intra-compartmental pressure). The exclusion criteria were: 1) a history of smoking; 2) a history of comorbidities that may affect inflammation, such as infections, autoimmune diseases, diabetes, and heart failure; and 3) a history of lower extremity fractures. Healthy subjects were recruited if they met the following inclusion criteria: 1) 18–65 years old; 2) without a history of lower extremity fractures and smoking; and 3) without a history of comorbidities that may affect inflammation, such as infections, autoimmune diseases, diabetes, and heart failure.

### Serum sample collection

We collected 5 ml of peripheral venous blood from 15 healthy subjects and 30 tibial fracture patients into tubes. The serum was then extracted by centrifugation at 3,000 rpm for 15 min and stored at −80°C until further analysis.

### Analysis of inflammation-related proteins

The Olink® target 92 inflammation panel (Olink Proteomics, LC-Bio Technology Co., Ltd. Hangzhou, China) was used to quantify protein levels according to the manufacturer’s instructions. Normalized Protein Expression (NPX), an arbitrary unit on the Log2 scale, was used to evaluate present protein abundance. A high protein level means a high NPX value. However, it is unable to compare NPX values between different proteins. The R package “OlinkAnalyze” was used to find differentially expressed proteins (DEPs) between groups. Principal component analysis (PCA), which highlights the most important aspects of data variability, was performed using the “princomp” function in R software. We used ggplot2 to generate heat map and volcano plots. Gene Ontology (GO) terms and Kyoto Encyclopedia of Genes and Genomes (KEGG) pathways were used to create the gene sets. Additionally, correlation analysis of the expression of two proteins was performed using Spearman’s correlation. The protein-protein interaction (PPI) network of the DEP was constructed and visualized by Cytoscape (version 3.9.1).

### ELISA validation

ELISA analysis was carried out with sera from 15 healthy subjects and 30 tibial fracture patients using a cysteine-cysteine motif chemokine ligand 20 (CCL23), interleukin-6 (IL-6), macrophage colony-stimulating factor (CSF-1), and hepatocyte growth factor (HGF) ELISA kit (MB-0074A, Jiangsu Meibiao Biological Technology, China) based on the manufacturer’s protocol.

### Statistical analysis

We utilized SPSS (version 25.0 SPSS Inc., Chicago, IL) and R software (version 4.2) with a P-value cutoff of 0.05. Regarding continuous variables, if measurement data met normality criteria, they were presented as mean ± SD (standard deviation) using a t-test or ANOVA, but if not, the Mann–Whitney U test was used to perform statistical analysis between groups. For count data, the chi-square test was used. Furthermore, we used receiver operating characteristic (ROC) curves to identify cutoff values for continuous variables. The area under the ROC curve (AUC) was used to determine the diagnostic ability, ranging from 0% to 100%, with more area meaning better ability. We chose the cutoff values for continuous variables using the maximum Youden index (sensitivity+specifcity-1) in the ROC curve analysis.

## Results

### Characteristics of healthy subjects and patients

**Figure 1:**
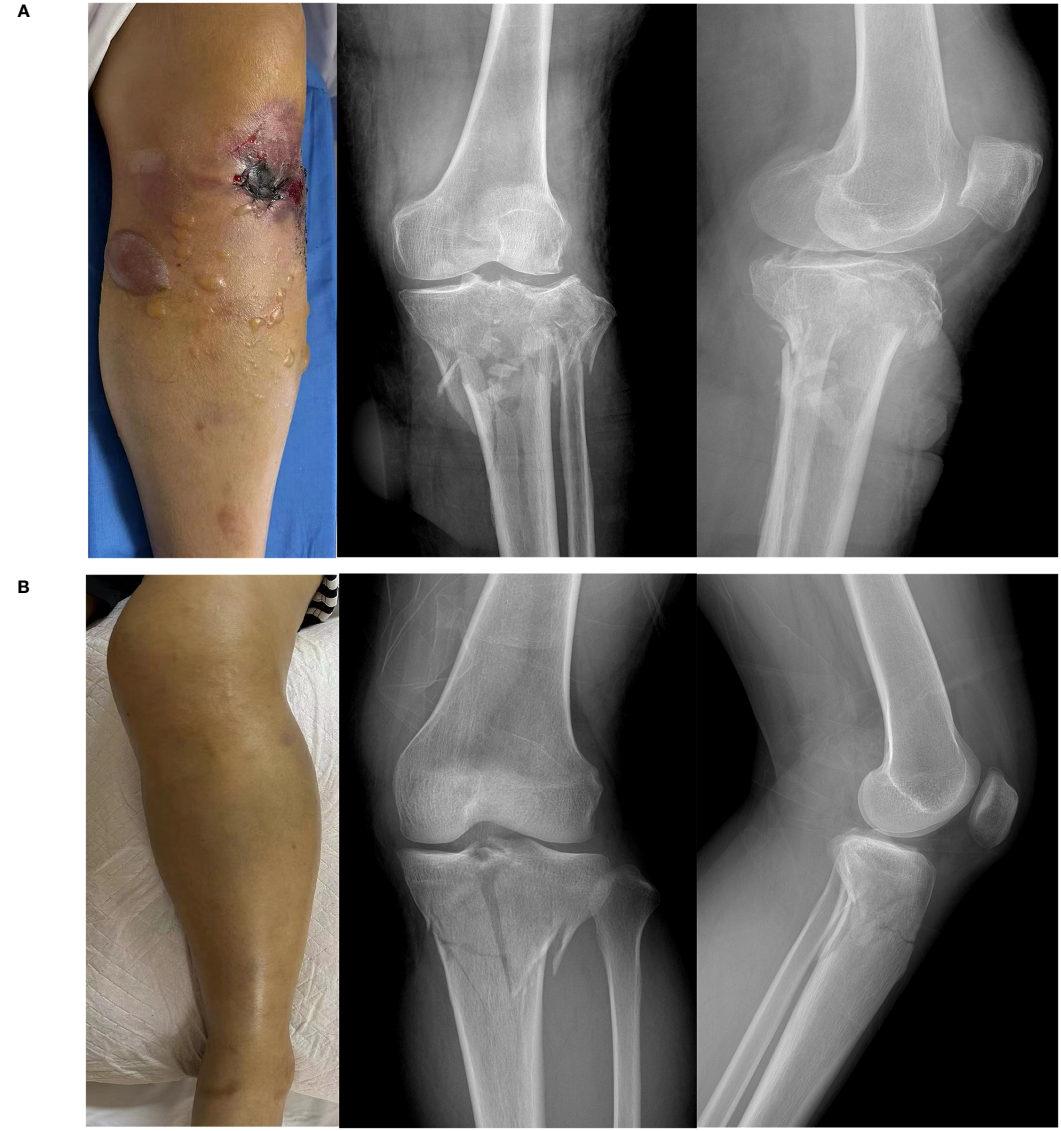
Images and X-ray of a tibial fracture. **(A)** Tibial fracture patient with acute compartment syndrome. **(B)** Tibial fracture patient without acute compartment syndrome.

Fifteen healthy subjects and 30 patients with tibial fractures, comprising 15 patients with ACS (**Figure 1A**) and 15 patients without ACS (**Figure 1B**), were included in our study. **Table 1** shows the characteristics of healthy subjects and patients with tibial fractures. There were no statistical differences in age, sex, or BMI between these groups.

**Table 1:** Characteristics of the human samples in the three groups.

Characteristic	CG (n=15)	AG (n=15)	FG (n=15)	p
**Age, years**	44.0 (34.0-51.0)	51.0 (37.0-56.0)	48.0 (36.0-57.0)	0.503
**Gender**				0.537
Male	8	9	6	
Female	7	6	9	
**Body mass index (kg/m2)**	24.7 (20.8-27.2)	23.4 (21.2-27.2)	24.2 (21.1-26.7)	0.844

CG, control group; AG, acute compartment syndrome group; FG, fracture group.

**Figure 2:**
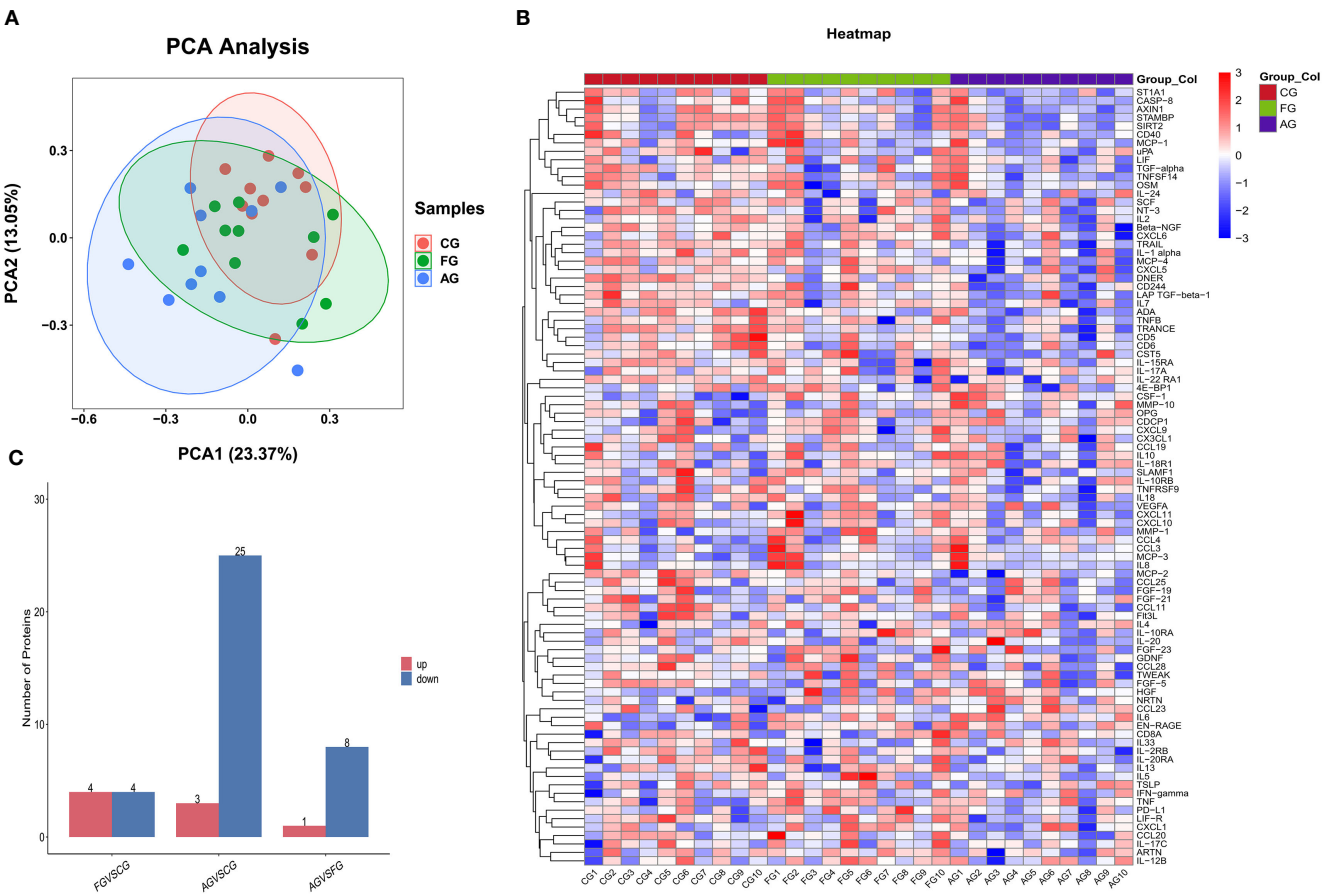
Comparison of the serum inflammation-related proteins between three groups. **(A)** Principal component analysis (PCA) showed two dimensions among three groups differentiated by color. Each point represents a single patient, with patients of similar protein expression profiles positioned next to each other. **(B)** Heat map of protein expression among three groups. The x-axis shows the sample group, and the y-axis shows the protein name. Different colors represent different levels of protein expression, with blue to red representing low to high levels of expression. **(C)** Comparison of the number of differential proteins between paired groups. CG, control group; AG, acute compartment syndrome group; FG, tibial fracture group.

We used PCA to investigate the similarity among the three groups. Regarding variance, 23.37% was explained by the first component, and 13.05% was explained by the second (**Figure 2A**). **Figure 2B** shows protein expression among the three groups using a heat map. Additionally, we found the number of upregulated and downregulated differential proteins between pairwise comparisons (**Figure 2C**).

### Comparison between the fracture and control groups

**Figure 3:**
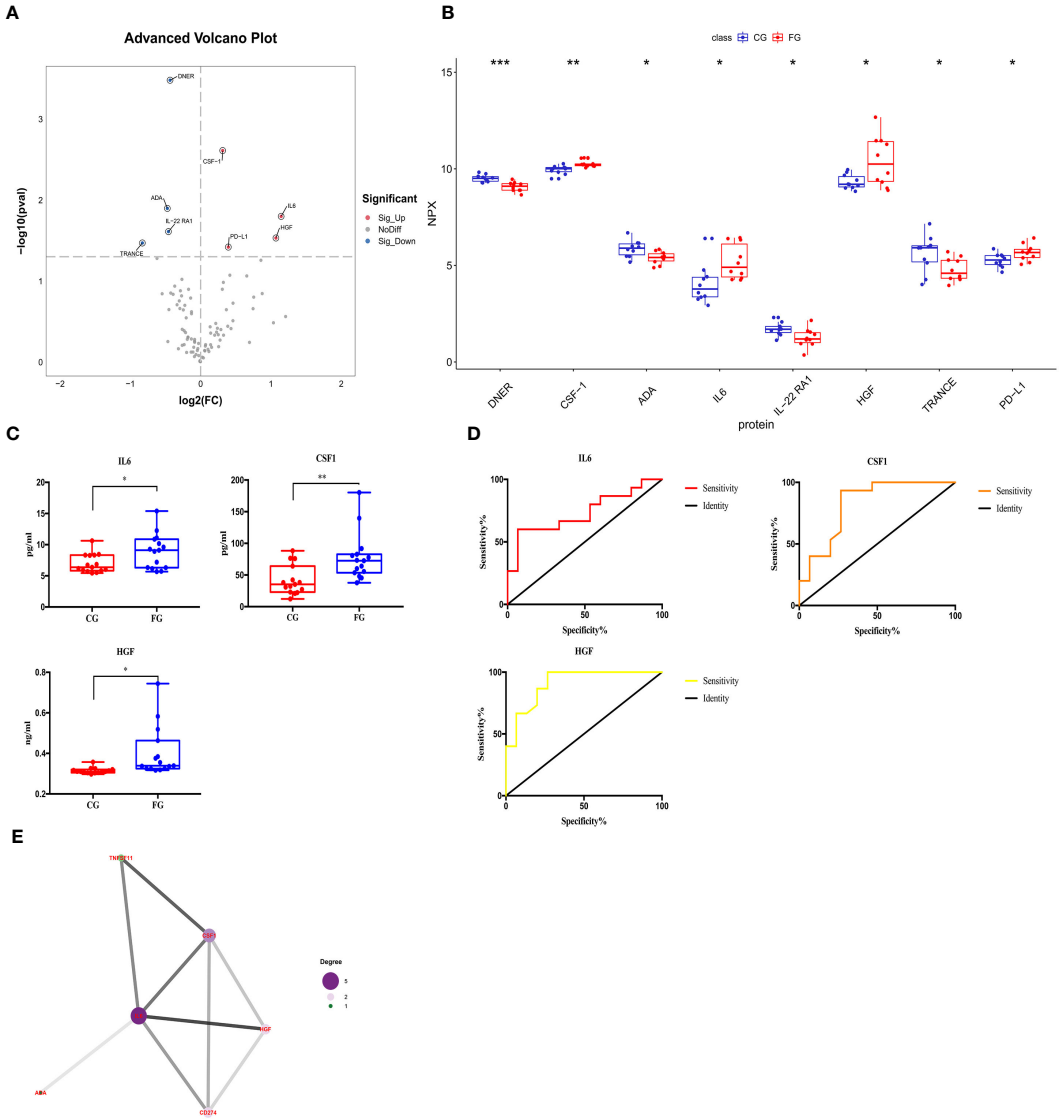
Comparison of the serum inflammation-related proteins between tibial fracture group (FG) and control group (CG). **(A)** Volcano plots showed differentially expressed proteins between the FG and CG. Proteins highly expressed in the FG and CG are labeled red and blue, respectively. Differences between the FG and CG were expressed as Log2 (fold change) of serum on the x-axis and the (-Log10) p value on the y-axis. **(B)** Box plot representing differentially expressed proteins between the FG and CG. **(C)** IL6, CSF-1, and HGF levels between the FG and CG, as determined by an ELISA, are shown in the box plot. **(D)** Receiver operating characteristic curve analysis of IL6, CSF-1, and HGF between the FG and CG. **(E)** Protein-protein interaction. CG, control group; FG, tibial fracture group. *<0.05, **<0.01, ***<0.001.

We compared the expression levels of 92 inflammation-related proteins between the FG and CG by Olink analysis, as shown in **Table 2**. We found eight DEGs in the FG group when compared with the CG; four were downregulated DEPs (DNER, ADA, IL-22RA1, and TRANCE) and four were upregulated DEPs (CSF-1, IL6, HGF, and PD-L1) (**Figures 2C**, **3A, B**). We used an ELISA to verify their expression levels and found that they were significantly higher in the FG than in the CG (all p < 0.05, **Figure 3C**). Furthermore, the ROC curve analysis showed the AUC of CSF-1 (*p*=0.0025, AUC area=0.8244, 95% CI [0.6691−0.9797]), IL6 (*p*=0.0294, AUC area=0.7333, 95% CI [0.5487−0.9179]), and HGF (*p*=0.0001, AUC area=0.9089, 95% CI [0.8043−1.000]) (**Figure 3D**, **Table 3**). Their optimal cutoff values based on the Youden index were 43.845 pg/ml, 8.69 pg/ml, and 0.3166 ng/ml, respectively (**Table 3**). Additionally, we found the combination with the highest diagnostic accuracy (*p*<0.0001, AUC area=0.951, 95% CI [0.882, 1.000]) (**Table 3**). Furthermore, we tried to investigate the interactions of DEPs, and the PPI network showed IL6 as the core protein (**Figure 3E**).

**Table 2:** Protein expression analysis among three groups.

Protein	Groups			P value		
	CG (n=10)	AG (n=10)	FG (n=10)	FG vs. CG	AG vs. CG	AG vs. FG
**4E-BP1**	6.45	6.19	6.71	0.64	0.63	0.41
**ADA**	5.87	5.06	5.39	0.01	0.00	0.11
**ARTN**	0.61	0.47	0.68	0.61	0.33	0.08
**AXIN1**	3.18	2.31	3.05	0.75	0.03	0.07
**Beta-NGF**	1.97	1.78	1.94	0.59	0.01	0.04
**CASP-8**	3.18	2.45	2.98	0.76	0.26	0.38
**CCL11**	8.07	7.18	7.73	0.20	0.00	0.03
**CCL19**	10.96	10.62	10.85	0.75	0.35	0.49
**CCL20**	8.20	7.62	8.48	0.53	0.06	0.07
**CCL23**	12.09	12.40	11.96	0.52	0.20	0.02
**CCL25**	6.38	5.98	5.99	0.15	0.20	0.97
**CCL28**	2.29	1.92	2.28	0.98	0.10	0.12
**CCL3**	6.77	7.34	7.52	0.22	0.32	0.79
**CCL4**	7.37	7.76	7.76	0.36	0.26	1.00
**CD244**	5.31	4.95	5.28	0.83	0.01	0.05
**CD40**	11.41	11.10	11.56	0.66	0.34	0.09
**CD5**	5.82	5.23	5.55	0.15	0.01	0.04
**CD6**	5.52	4.67	5.09	0.14	0.00	0.14
**CD8A**	9.91	10.04	10.02	0.74	0.65	0.96
**CDCP1**	3.50	3.87	3.83	0.47	0.42	0.90
**CSF-1**	9.95	10.30	10.26	0.00	0.00	0.60
**CST5**	7.33	6.93	7.23	0.66	0.05	0.22
**CX3CL1**	4.21	4.03	4.12	0.53	0.29	0.54
**CXCL1**	9.67	9.70	10.08	0.08	0.92	0.25
**CXCL10**	8.64	8.57	9.10	0.31	0.85	0.21
**CXCL11**	8.14	7.61	9.01	0.14	0.15	0.03
**CXCL5**	12.37	11.59	12.62	0.46	0.13	0.07
**CXCL6**	9.07	8.54	9.18	0.70	0.11	0.07
**CXCL9**	6.86	6.80	7.15	0.40	0.80	0.24
**DNER**	9.51	8.96	9.07	0.00	0.00	0.48
**EN-RAGE**	6.36	7.07	7.11	0.16	0.22	0.92
**FGF-19**	8.76	8.55	9.19	0.39	0.69	0.20
**FGF-21**	4.58	4.04	4.31	0.62	0.32	0.50
**FGF-23**	0.92	1.07	1.38	0.19	0.55	0.44
**FGF-5**	2.05	1.96	1.90	0.16	0.50	0.64
**Flt3L**	9.16	8.89	8.92	0.41	0.44	0.90
**GDNF**	2.26	2.02	2.32	0.73	0.04	0.09
**HGF**	9.33	10.37	10.40	0.03	0.00	0.96
**IFN-gamma**	6.97	7.48	7.82	0.06	0.23	0.41
**IL-1 alpha**	0.66	0.25	0.37	0.13	0.04	0.38
**IL10**	2.97	3.13	3.28	0.13	0.46	0.49
**IL-10RA**	1.18	1.24	1.42	0.38	0.81	0.56
**IL-10RB**	6.25	6.16	6.22	0.68	0.44	0.60
**IL-12B**	6.20	6.28	6.57	0.23	0.82	0.38
**IL13**	0.68	0.31	0.50	0.49	0.12	0.23
**IL-15RA**	1.61	1.47	1.48	0.25	0.08	0.97
**IL-17A**	1.25	1.14	1.47	0.43	0.55	0.24
**IL-17C**	2.54	2.48	2.73	0.46	0.79	0.27
**IL18**	9.63	9.13	9.33	0.22	0.05	0.35
**IL-18R1**	7.36	7.38	7.42	0.72	0.91	0.85
**IL2**	1.16	0.97	1.07	0.57	0.13	0.53
**IL-20**	1.22	1.14	1.09	0.24	0.62	0.80
**IL-20RA**	1.23	1.08	1.18	0.72	0.24	0.47
**IL-22 RA1**	1.71	1.19	1.25	0.02	0.01	0.77
**IL-24**	1.75	1.64	1.53	0.20	0.51	0.53
**IL-2RB**	0.86	0.55	0.59	0.10	0.05	0.69
**IL33**	1.67	1.58	1.50	0.13	0.34	0.48
**IL4**	0.46	0.44	0.60	0.44	0.92	0.46
**IL5**	1.20	0.84	2.23	0.33	0.09	0.19
**IL6**	4.04	5.73	5.18	0.02	0.00	0.23
**IL7**	3.03	2.41	2.84	0.52	0.06	0.18
**IL8**	7.71	7.61	8.40	0.57	0.93	0.55
**LIF**	0.75	0.70	0.81	0.63	0.67	0.43
**LIF-R**	3.31	3.12	3.33	0.87	0.18	0.11
**MCP-1**	12.45	12.02	12.44	0.98	0.24	0.41
**MCP-2**	10.42	9.11	9.80	0.05	0.01	0.07
**MCP-3**	3.22	4.00	4.43	0.27	0.47	0.71
**MCP-4**	15.85	14.60	15.40	0.22	0.01	0.10
**MMP-1**	15.45	15.69	16.10	0.09	0.48	0.27
**MMP-10**	8.20	8.39	8.14	0.81	0.57	0.40
**NRTN**	0.79	1.08	0.92	0.37	0.08	0.39
**NT-3**	3.23	2.68	2.92	0.18	0.01	0.27
**OPG**	9.99	10.21	10.21	0.33	0.33	0.99
**OSM**	7.30	6.86	6.99	0.68	0.47	0.88
**PD-L1**	5.27	5.23	5.66	0.04	0.82	0.03
**SCF**	9.70	9.29	9.49	0.43	0.12	0.54
**SIRT2**	2.93	2.25	2.84	0.85	0.14	0.23
**SLAMF1**	2.18	2.01	2.17	0.96	0.35	0.25
**ST1A1**	4.14	2.66	3.67	0.56	0.04	0.18
**STAMBP**	4.17	3.50	3.90	0.39	0.04	0.23
**TGF-α**	5.33	4.66	4.79	0.14	0.04	0.77
**TGF-β-1**	7.41	6.64	7.04	0.12	0.01	0.16
**TNF**	3.54	3.68	3.87	0.10	0.48	0.29
**TNFB**	4.64	4.07	4.46	0.49	0.01	0.16
**TNFRSF9**	6.11	5.63	5.91	0.28	0.02	0.14
**TNFSF14**	6.75	6.01	6.52	0.61	0.11	0.32
**TRAIL**	9.00	8.44	8.91	0.55	0.01	0.03
**TRANCE**	5.61	4.08	4.78	0.03	0.00	0.05
**TSLP**	0.70	0.46	0.69	0.99	0.19	0.16
**TWEAK**	10.02	9.66	10.06	0.89	0.16	0.26
**uPA**	10.64	10.35	10.40	0.23	0.15	0.78
**VEGFA**	12.75	12.62	12.89	0.61	0.70	0.42

CG, control group; AG, acute compartment syndrome group; FG, fracture group.

**Table 3:** ROC curve analysis and cutoff value between paired groups.

Variables	Area	P-value	95% CI		Cutoff value
			Lower limit	Upper limit	
AG vs. FG					
CCL23	0.848	0.0011	0.7074	0.9904	35.75 pg/ml
AG vs. CG					
IL6	0.82	0.0028	0.6722	0.9678	9.225 pg/ml
CSF1	0.9689	<0.0001	0.9055	1.000	81.04 pg/ml
HGF	0.9867	<0.0001	0.9552	1.000	0.3301 ng/ml
IL6+CSF1	0.991	<0.0001	0.968	1.000	NA
IL6+HGF	0.996	<0.0001	0.981	1.000	NA
CSF1+HGF	1.000	<0.0001	1.000	1.000	NA
IL6+CSF1+HGF	1.000	<0.0001	1.000	1.000	NA
FG vs. CG					
IL6	0.7333	0.0294	0.5487	0.9179	8.69 pg/ml
CSF1	0.8244	0.0025	0.6691	0.9797	43.845 pg/ml
HGF	0.9089	0.0001	0.8043	1.000	0.3166 ng/ml
IL6+CSF1	0.849	0.001	0.715	0.983	NA
IL6+HGF	0.924	<0.0001	0.829	1.000	NA
CSF1+HGF	0.938	<0.0001	0.851	1.000	NA
IL6+CSF1+HGF	0.951	<0.0001	0.882	1.000	NA

CG, control group; AG, acute compartment syndrome group; FG, fracture group.

**Figure 4:**
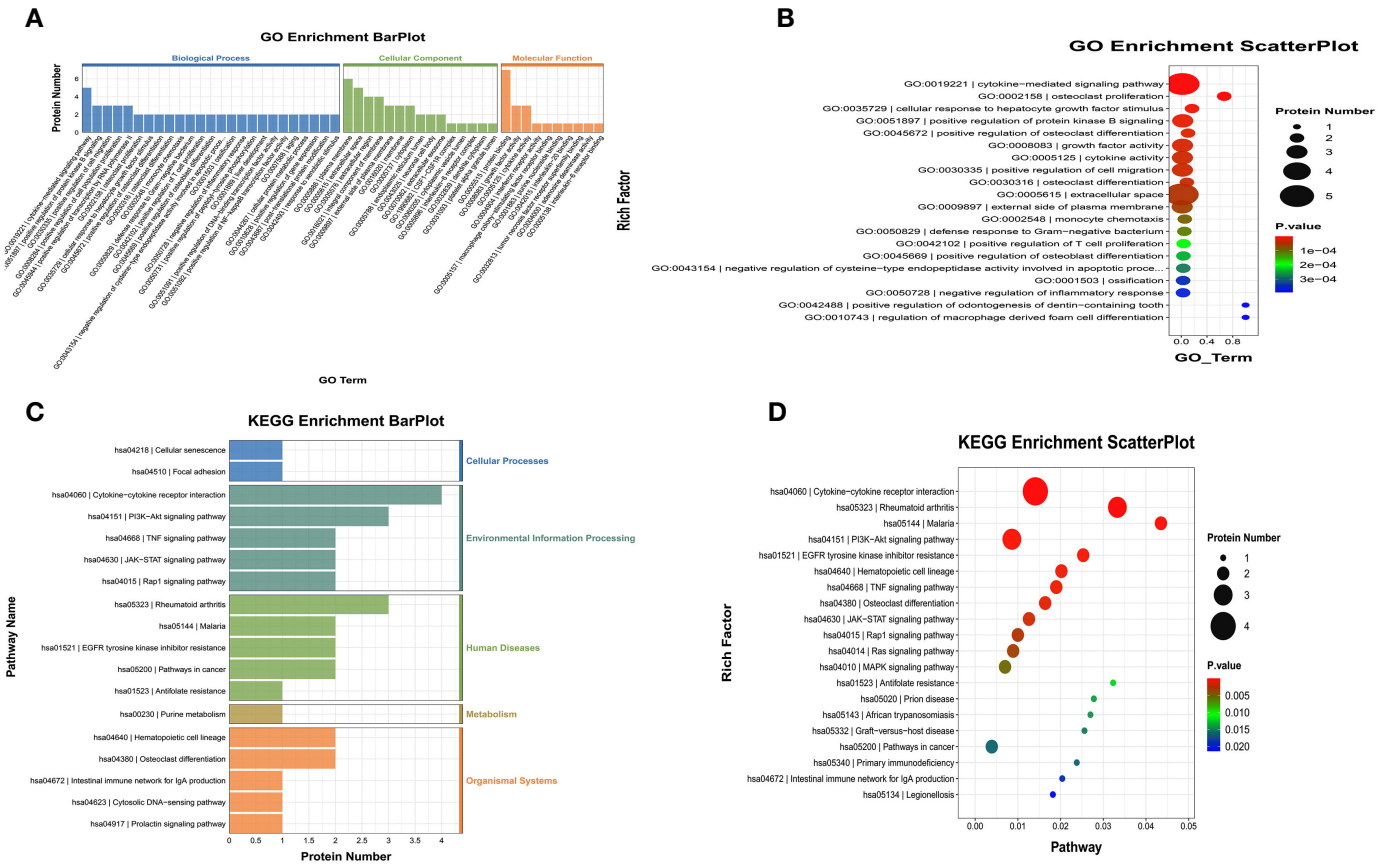
Gene Ontology (GO) terms and Kyoto Encyclopedia of Genes and Genomes (KEGG) of the significant inflammation proteins between the FG and CG. **(A, B)** GO terms of the significant inflammation proteins between the FG and CG. **(C, D)**. KEGG enrichment analysis of the significant inflammation proteins between the FG and CG. CG, control group; FG, tibial fracture group.

We also performed GO and KEGG enrichment analyses to investigate the potential functions of DEPs in the FG. In the GO enrichment analysis, the results indicated that the cytokine-mediated signaling pathway, cellular response to hepatocyte growth factor stimulus, and cytokine activity were enriched (**Figures 4A, B**). Furthermore, KEGG enrichment analysis implied that cytokine-cytokine receptor interaction, the PI3K-Akt signaling pathway, and the TNF signaling pathway were enriched (**Figures 4C, D**).

### Comparison between the ACS and control groups

**Figure 5:**
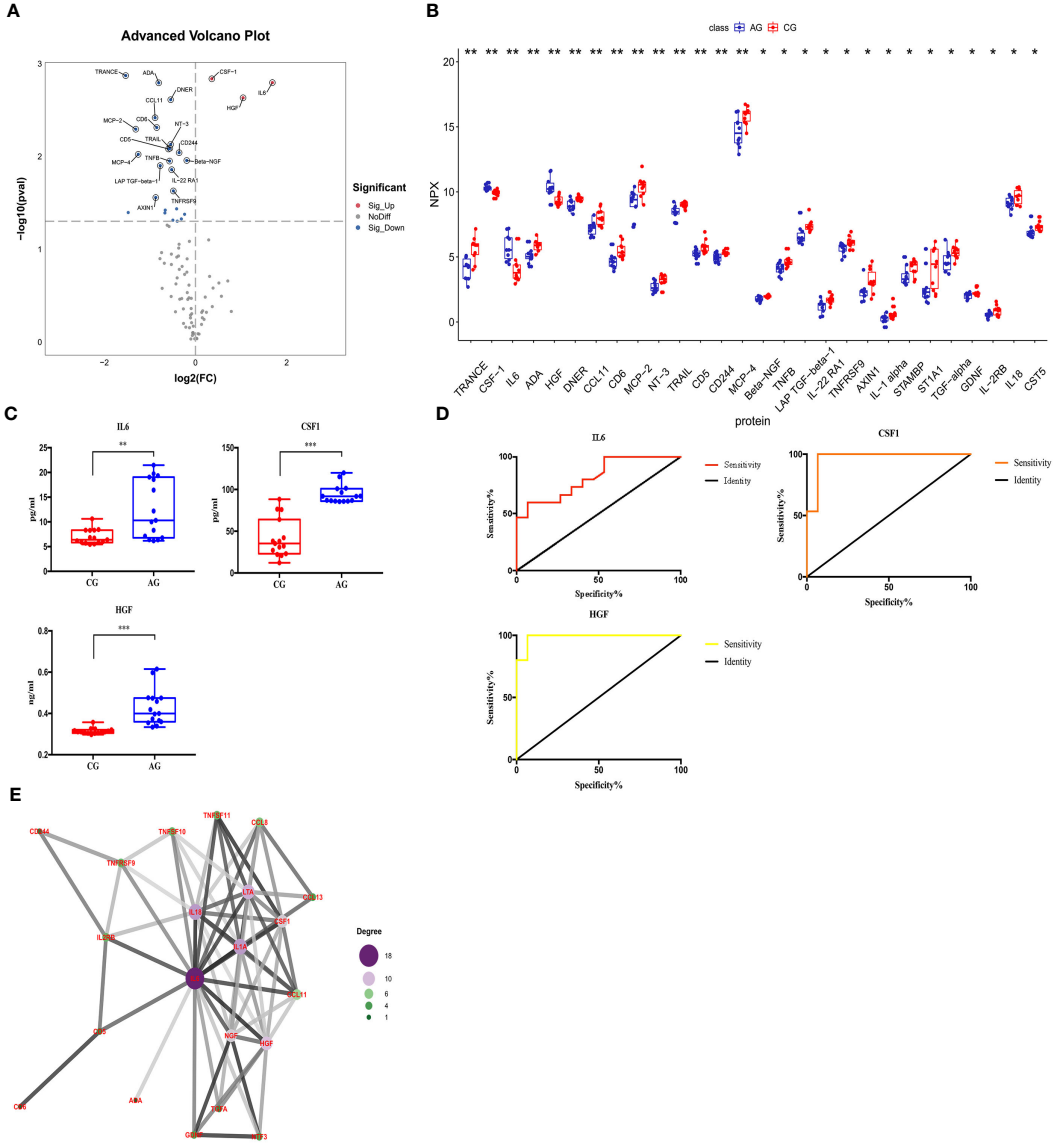
Comparison of the serum inflammation-related proteins between the acute compartment syndrome group (AG) and control group (CG). **(A)** A volcano plot shows the differentially expressed proteins between the AG and CG. Proteins highly expressed in the AG and CG are labeled in red and blue, respectively. Differences between the AG and CG are expressed as Log2 (fold change) of plasma on the x-axis and the (-Log10) p value on the y-axis. **(B)** Box plot representing differentially expressed proteins between the AG and CG. **(C)** IL6, CSF-1, and HGF levels between the AG and CG, as determined by an ELISA, are shown in the box plot. **(D)** Receiver operating characteristic curve analysis of IL6, CSF-1, and HGF between the AG and CG. **(E)** Protein-protein interaction. CG, control group; AG, acute compartment syndrome group. *<0.05, **<0.01, ***<0.001.

We compared the expression levels of 92 inflammation-related proteins between the AG and CG by Olink analysis, as shown in **Table 2**. We found 28 DEPs in the AG compared with the CG; 25 DEPs were downregulated and three were upregulated (CSF-1, IL6, and HGF) (**Figures 2C**, **5A, B**). ELISA validation indicated that their expression levels were significantly higher in the AG than in the CG (all p < 0.05, **Figure 5C**). Furthermore, the ROC curve showed the AUC of CSF-1 (*p*<0.0001, AUC area=0.9689, 95% CI [0.9055−1.000]), IL6 (*p*=0.0028, AUC area=0.82, 95% CI [0.6722−0.9678]), and HGF (*p*<0.0001, AUC area=0.9867, 95% CI [0.9552−1.000]) (**Figure 5D**; **Table 3**). Their optimal cutoff values were 81.04 pg/ml, 9.225 pg/ml, and 0.3301 ng/ml, respectively (**Table 3**). Additionally, we found the combination with the highest diagnostic accuracy (*p*<0.0001, AUC area=1.000, 95% CI [1.000, 1.000]) (**Table 3**). Furthermore, we tried to investigate the interactions of DEPs, and the PPI network showed IL6 as the core protein (**Figure 5E**).

**Figure 6:**
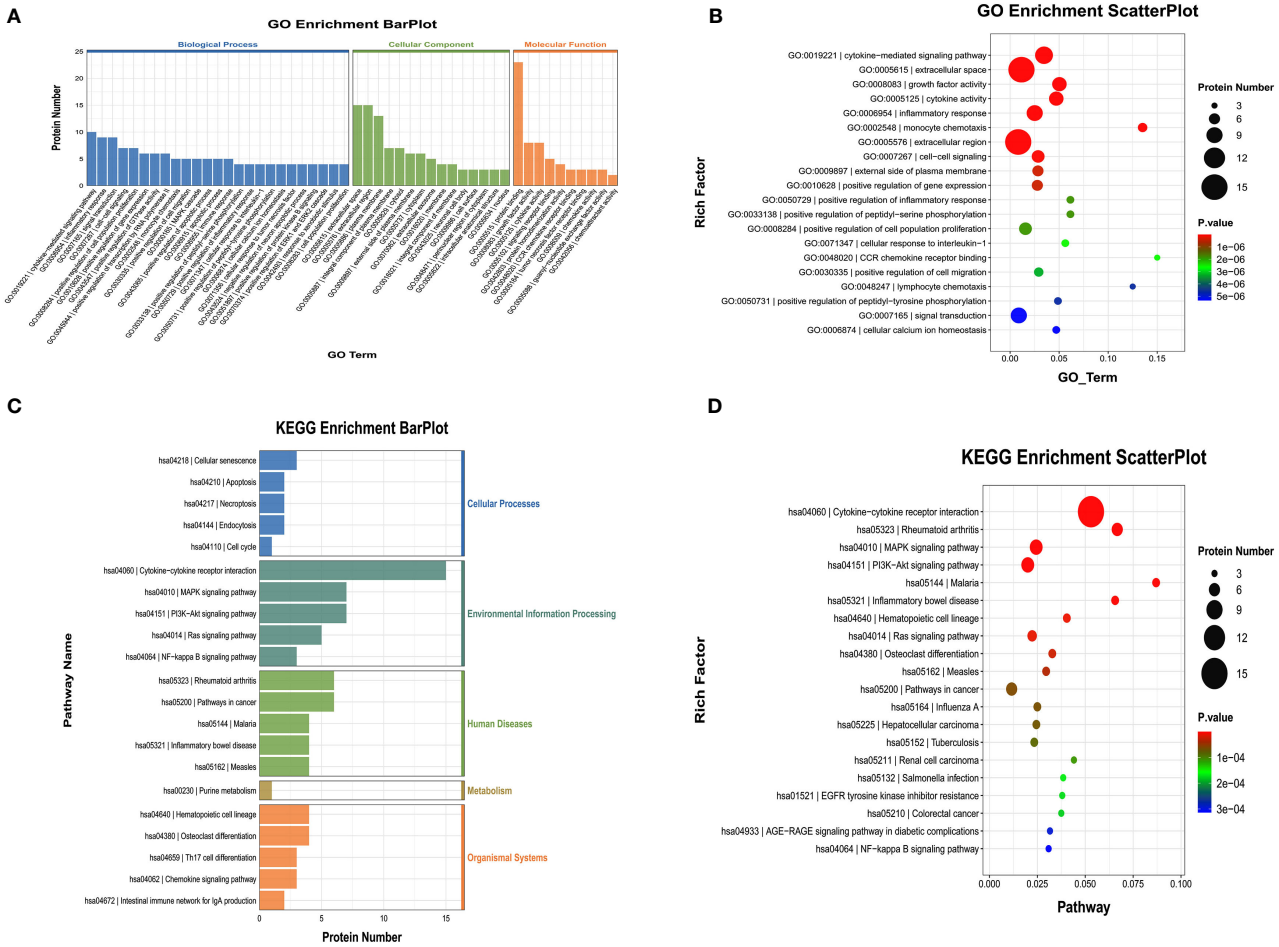
Gene Ontology (GO) terms and Kyoto Encyclopedia of Genes and Genomes (KEGG) of the significant inflammation proteins between the AG and CG. **(A, B)** GO terms of the significant inflammation proteins between the AG and CG. **(C, D)** KEGG enrichment analysis of the significant inflammation proteins between the AG and CG. CG, control group; AG, acute compartment syndrome group.

We also performed GO and KEGG enrichment analyses to investigate the potential functions of DEPs in the AG. In GO enrichment analysis, the results indicated that the cytokine-mediated signaling pathway, inflammatory response, and cytokine activity were enriched (**Figures 6A, B**). Furthermore, KEGG enrichment analysis implied that cytokine-cytokine receptor interaction, the PI3K-Akt signaling pathway, and the MAPK signaling pathway were enriched (**Figures 6C, D**).

### Comparison between the ACS and fracture groups

**Figure 7:**
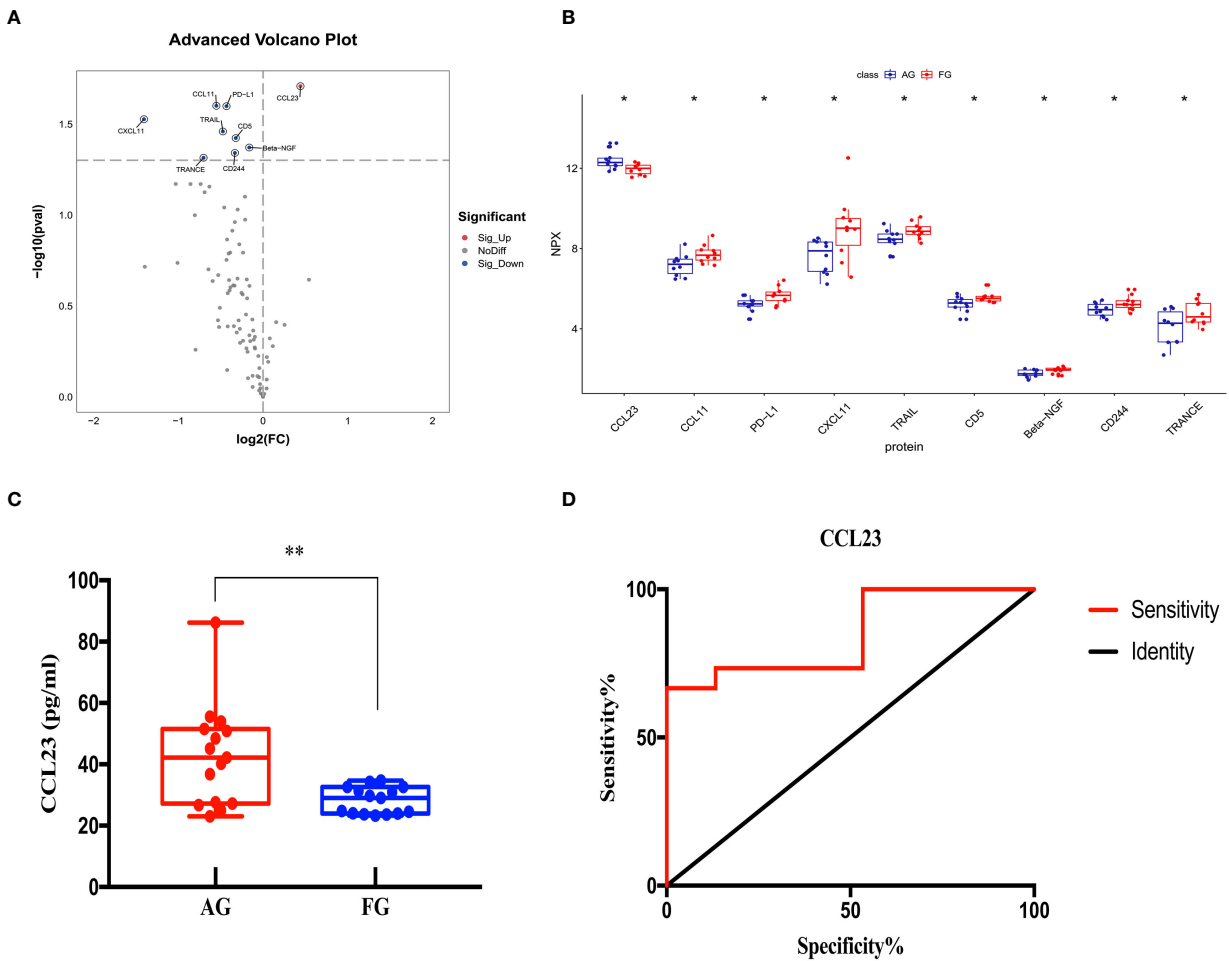
Comparison of the serum inflammation-related proteins between the acute compartment syndrome group (AG) and tibial fracture group (FG). **(A)** A volcano plot shows differentially expressed proteins between the AG and FG. Proteins highly expressed in the AG and FG are labeled in red and blue, respectively. Differences between the AG and FG are expressed as Log2 (fold change) of plasma on the x-axis and the (-Log10) p value on the y-axis. **(B)** Box plot representing differentially expressed proteins between the AG and FG. **(C)** The CCL23 level between the AG and FG, as determined by an ELISA, is shown in the box plot. **(D)** Receiver operating characteristic curve analysis of CCL23 between the AG and FG. AG, acute compartment syndrome group; FG, tibial fracture group. *<0.05, **<0.01.

We compared the expression levels of 92 inflammation-related proteins between the AG and FG by Olink analysis, as shown in **Table 2**. We found nine DEPs in the AG group compared with the FG; eight DEPs were downregulated and one was upregulated (CCL23) (**Figures 2C**, **7A, B**). ELISA validation showed that the expression level of CCL23 was significantly higher in the AG than in the FG (P < 0.05, **Figure 7C**). Furthermore, the ROC curve analysis showed the AUC of CCL23 (*p*=0.0011, AUC area=0.848, 95% CI [0.7074−0.9904]) (**Figure 7D**; **Table 3**). Its optimal cutoff value was 35.75 pg/ml (**Table 3**).

**Figure 8:**
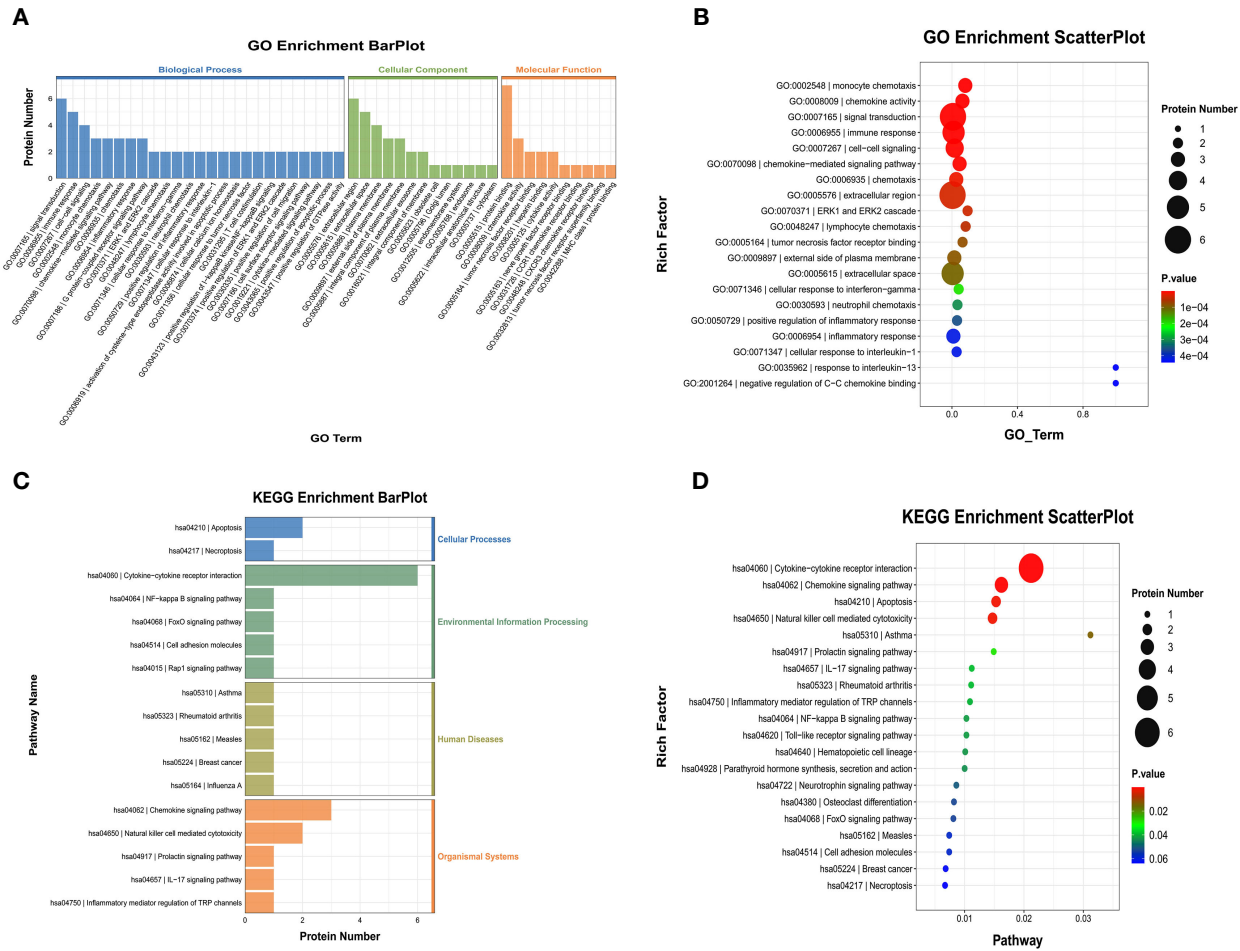
Gene Ontology (GO) terms and Kyoto Encyclopedia of Genes and Genomes (KEGG) of the significant inflammation proteins between the AG and FG. **(A, B)** GO terms of the significant inflammation proteins between the AG and FG. **(C, D)** KEGG enrichment analysis of the significant inflammation proteins between the AG and FG. CG, control group; FG, tibial fracture group.

We also performed GO and KEGG enrichment analyses to investigate the potential functions of DEPs in the AG. In GO enrichment analysis, the results indicated that monocyte chemotaxis, chemokine activity, immune response, inflammatory response, and the cytokine-mediated signaling pathway were enriched (**Figures 8A, B**). Furthermore, KEGG enrichment analysis implied that cytokine-cytokine receptor interaction and the chemokine signaling pathway were enriched (**Figures 8C, D**).

## Discussion

Despite a substantial improvement in its management, there is a sustainable need for reliable and rapid methods to diagnose ACS and improve its therapeutic efficacy, which may influence patient outcomes. The need for a fast interventional step is especially important because ACS may cause muscle necrosis, function deficit, or amputation (1, 2, 7). Notably, there is no available biomarker of ACS with high specificity, which presents an enormous challenge for orthopedic surgeons in diagnosing it without delay. In our previous research, complete blood counts revealed that the levels of NEU and CKMB were related to ACS, but a limited number of inflammation-related indicators were observed. Our recent studies also found some variations of inflammatory cytokines in patients with fracture blister fluid and plasma through Olink Proteomics analysis (11, 12). Therefore, we used the inflammatory panel of Olink Proteomics analysis to identify the biomarkers of ACS in the current study.

Our findings showed that the levels of IL6, CSF-1, and HGF in the FG were significantly higher than those in the CG. Similar results were found between the AG and CG, and their cutoff values for predicting ACS compared with the CG were 9.225 pg/ml, 81.04 pg/ml, and 0.3301 ng/ml, respectively. Furthermore, their combination had the highest diagnostic accuracy. Notably, compared with the FG, we only found a higher expression of CCL23 in the AG. Additionally, we identified 35.75 pg/ml as the cutoff value of CCL23 for predicting ACS in patients with tibial fractures.

CCL23, also known as myeloid progenitor inhibitory factor 1 (MPIF1) or macrophage inflammatory protein 3, is a crucial chemokine that has been reported to be involved in the progression of the inflammatory response by recruiting immune cells, such as monocytes, macrophages, dendritic cells, and lymphocytes, and directly improving their migration to the sites of injury or infection (13, 14). Circulating CCL23 upregulates several adhesion molecules that promote the migration of circulating immune cells to the inflammatory microenvironment after interacting with CC chemokine receptor 1 (14). Activating the CCL23-CCR1 axis also irritates the release of other pro-inflammatory cytokines, including macrophage inflammatory protein-1α, interleukin-1β, and tumor necrosis factor-α (15), and stimulates angiogenesis via the promotion of endothelial cell migration through the upregulation of several matrix metalloproteinases in the endothelium.

Substantial evidence indicates that the blood level of CCL23 can reflect the progression of several inflammatory diseases, including atherosclerosis, systemic mastocytosis, systemic sclerosis, acute myeloid leukemia, and chronic kidney disease (16–18). Additionally, the level of circulating CCL23 has been associated with markers of atheroprogression, such as aortic wall thickness and plaque burden (14). However, the relationship between CCL23 and ACS remains obscure. We only found that the level of CCL23 was significantly higher in the AG than in the FG, which was partially consistent with our previous conclusions that there was a significant variation in monocytes and macrophages in patients with ACS (19). Furthermore, we identified 35.75 pg/ml as the cutoff value of CCL23 for predicting ACS in tibial fracture patients. This implies that a serum level of CCL23 is a potential marker of ACS differentiating from tibial fractures. However, only one of the 92 inflammatory-related proteins was found to be significantly elevated, which may imply that there is not much difference between the two groups regarding inflammation. Therefore, we need to expand the sample size for further research.

IL-6 is a well-known and important immunomodulatory cytokine that is involved in the process of numerous diseases, including autoimmune diseases and chronic inflammatory conditions (20), and is considered a diagnostic tool and potential therapeutic target for various injuries, such as traumatic brain injury, post-infarction cardiac injury, and acute kidney injury (20–22). CSF-1 has been reported to promote the differentiation of myeloid progenitors into monocytes, macrophages, and dendritic cells. It governs the migration, proliferation, activity, and survival of macrophages in the periphery, which has a variety of roles in both the innate and adaptive immune systems. Various diseases, such as cancer, inflammation, and bone disease, are linked to and made worse by the macrophage populations that are stimulated by CSF-1. On the other hand, macrophages are involved in tissue healing, disease eradication, and immunosuppression (23, 24). HGF and its specific receptor, mesenchymal-epithelial transition factor (c-Met), are widely expressed in numerous cell types, such as endothelial cells, vascular smooth muscle cells, cardiomyocytes, and anti-inflammatory M2 macrophages (25). Regarding anti-inflammatory signaling pathways, HGF/c-Met recently attracted more attention because it contributed to the restoration of the impaired resolution of inflammation and regulation of inflammatory immune cells (26).

Interestingly, we found significantly higher expression of CSF-1, IL6, and HGF in both the AG and FG in comparison with the CG, implying that, to some extent, the immune responses caused by the two groups are similar. Our findings also showed that IL6 is the core protein. It is well-accepted that CSF-1, IL6, and HGF play crucial roles in macrophage polarization. IL6 contributes to the regulation of macrophages toward M1 macrophage polarization, whereas CSF-1 and HGF stimulate macrophages toward M2 macrophage polarization. Our previous single-cell RNA analysis showed that the proportion of macrophages has undergone significant changes, especially M1 macrophages and M2 macrophages, indicating that the balance of M1 macrophages and M2 macrophages may affect the development of ACS (19). We can assume that CSF-1, IL6, and HGF are involved in the development of ACS patients. Furthermore, we identified 9.225 pg/ml, 81.04 pg/ml, and 0.3301 ng/ml as the cutoff values of CSF-1, IL6, and HGF, respectively, for predicting ACS in healthy subjects, and we found their combination to have the highest diagnostic value. This implies that serum levels of CSF-1, IL6, and HGF are potential markers of ACS differentiating from healthy subjects.

Although, to our best knowledge, this is the first study to investigate the potential biomarkers of ACS by Olink Proteomics analyses, some limitations should be noted. First, owing to the relatively low rate of ACS, its small sample size may constrain the exactitude of our findings. A large-scale multicenter study should be conducted for further research. Second, we only focused on the potential role of inflammation-related indicators in the prediction of ACS and did not consider other possible factors, such as genetics and environmental factors. In the next step, we will fully consider these factors and carry out a more comprehensive clinical study. Third, owing to the difficulty of collecting samples, dynamic variations of relevant inflammatory markers could not be observed. Further well-designed prospective studies are needed to verify our results.

In summary, we identified CCL23 as a potential biomarker of ACS in comparison with tibial fracture patients and the significance of the combined diagnosis of CSF-1, IL6, and HGF for predicting ACS compared with healthy individuals. Furthermore, we also found their cutoff values and combinations with the highest diagnostic accuracy, providing clinicians with a new method for rapidly diagnosing ACS. In addition, more samples are needed to further validate our conclusion.

## Data availability statement 

The raw data supporting the conclusions of this article will be made available by the authors, without undue reservation.

## Ethics statement

The study was approved by the Institutional Review Board of the Third Hospital of Hebei Medical University before data collection and analysis. The studies were conducted in accordance with the local legislation and institutional requirements. The participants provided their written informed consent to participate in this study. Written informed consent was obtained from the individual(s), and minor(s)’ legal guardian/next of kin, for the publication of any potentially identifiable images or data included in this article.

## Author contributions

ZH: Supervision; Writing – review & editing. TaoW: Writing – original draft. SY: Writing – original draft. YRL: Conceptualization; Writing – original draft. YBL: Conceptualization; Investigation; Writing – review & editing. TingW: Validation; Writing – review & editing.
